# Single-Center Outcomes of WATCHMAN™ Implantation with Comparison to Oral Anticoagulant and Dual Antiplatelet Therapy

**DOI:** 10.7759/cureus.47444

**Published:** 2023-10-21

**Authors:** Francis Demiraj, Michael S Benrubi, Denis Babici, Eti Muharremi, Ronald Pachon, Ahmed Osman

**Affiliations:** 1 Department of Neurology, Florida Atlantic University Charles E. Schmidt College of Medicine, Marcus Neuroscience Institute, Boca Raton, USA; 2 Department of Neurology, Nova Southeastern University Dr. Kiran C. Patel College of Osteopathic Medicine, Boca Raton, USA; 3 Department of Neurology, Florida Atlantic University Charles E. Schmidt College of Medicine, Boca Raton, USA; 4 Department of Neurology, Columbia University Irving Medical Center, New York, USA; 5 Department of Cardiology, University of Miami, Miami, USA; 6 Department of Cardiology, Broward General Medical Center, Fort Lauderdale, USA

**Keywords:** atrial fibrillation, dual antiplatelet therapy (dapt), watchman device, prevention of ischemic stroke, direct oral anticoagulant therapy

## Abstract

Background

The WATCHMAN™ device is a Food and Drug Administration (FDA)-approved device that reduces the risk of stroke from atrial fibrillation (AF) in those who have a contraindication to taking oral anticoagulation. A key aspect of this device implantation is the choice of medical therapy in the months after device implantation with Vitamin K antagonist oral anticoagulants (OAC) being the mainstay of therapy but dual antiplatelet therapy (DAPT) poses as a potential alternative to patients who have a contraindication to OAC use.

Methods

Our single-center study retroactively followed 150 patients post-WATCHMAN™ implantation and evaluated outcomes at 12 months post-implantation in two cohorts, those treated with OAC or DAPT. Our results were obtained via chart review of a single-center electronic medical records system.

Results

In our study, 67.33% of study patients were males and 49.33% were on OAC compared to 36.00% that were on DAPT. Ten patients were not able to undergo device implantation. With this analysis, we found similarly low rates of complications such as stroke and device-associated thrombosis (DAT) in both groups. Our DAPT cohort did have a higher number of gastrointestinal (GI) bleeding but this was not significant in our analysis.

Discussion

Our study compares to larger trials that show similar outcomes between OAC and DAPT post-implantation of the WATCHMAN™ device. The increased number of GI bleeding in our DAPT cohort could be the result of the underlying advanced age and comorbidity of that patient cohort.

Conclusion

Our results suggest that DAPT is a safe alternative to OAC for patients undergoing WATCHMAN™ implantation.

## Introduction

Atrial fibrillation (AF) predisposes affected patients to an increased propensity to form clots via several mechanisms. In AF, the left atrium incompletely contracts, which causes a stasis of coagulation factors in the left atrial appendage. The WATCHMAN™ device addresses this pathology by serving to seal off the left atrial appendage and eliminate the opportunity for coagulation in this location of the heart. The WATCHMAN™ device is an FDA-approved device to reduce the risk of stroke from AF in those who have a contraindication to taking anticoagulation [1].

Based on the post-implantation treatment protocols from the PROTECT AF and PREVAIL trials, the vast majority of WATCHMAN™ implantations described in the literature were accompanied by warfarin anticoagulation for 45 days, followed by dual antiplatelet therapy (DAPT) for six months post-procedure and aspirin thereafter [2,3]. For patients who cannot tolerate oral anticoagulation (OAC) for 45 days post-procedure, DAPT therapy has been used for six months post-implantation as an alternative.

Currently, the optimal DAPT regimen and its duration after WATCHMAN™ implantation are still under debate and might be patient-specific. OAC remains the standard therapy in patients with low bleeding risk, whereas the use of antiplatelet agents may be indicated in some clinical settings when the risk of thromboembolism is balanced by the risk of bleeding [4]. The role of non-vitamin K antagonist oral anticoagulants (NOACs), which have seen increases in usage in recent years, is unknown.

The goal of our work was to retrospectively evaluate post-WATCHMAN™ implantation medication therapy and examine complication rates at 12 months post-implantation for our cohort. The DAPT regimen was evaluated in comparison to OAC use and special attention was given to the rate of device-associated thrombus (DAT), given the DAT rates shown in the PROTECT-AF, PREVAIL, CAP, and CAP2 trials [5].

## Materials and methods

This retrospective observational study evaluated 150 patients who underwent WATCHMAN™ implantation from November 2015 to May 2019. All procedures involving human participants were in accordance with the ethical standards of the institutional research committee, the 1964 Helsinki Declaration, and its later amendments or comparable ethical standards. The Broward Health IRB approved the conduct of this human study (approval number BRH00025709). Implantation drug regimens and outcomes were obtained from a retroactive chart review of a single health system's electronic medical record. The inclusion criteria for this study were patients who were implanted with the WATCHMAN™ device at the same single center and were followed in the same health system. All WATCHMAN™ procedures conducted in our study were performed by the same team of two cardiac electrophysiologists who performed device implantation via the standard femoral vein approach. All patients included in the study underwent transesophageal echocardiography (TEE) at 45 days and six months post-procedure. The choice of medical therapy after device implantation was based solely on the patient’s ability to tolerate OAC therapy. Patients who were not able to tolerate OAC were placed on DAPT. Common reasons for OAC intolerance were history of bleeding, previous adverse reactions to OAC, documented medication nonadherence, and increased occupational risk of being on an OAC. Patients in our OAC cohort received OAC for 45 days followed by DAPT until their six-month TEE. Patients in our DAPT cohort received DAPT from the time of implantation until their six-month TEE. All patients in the DAPT and OAC groups had successful implantation of the WATCHMAN™ device as visualized by their six-month TEE. No patient in these two groups required prolongation of their medical therapy. Both groups were placed on aspirin indefinitely after successful TEE at the six-month mark. Patients were excluded if their 12-month period monitoring was incomplete due to lack of follow or transfer to another health system. These patients were not part of the 150 included in the study and comprised a small portion (<10 percent) of those analyzed for inclusion. Evaluation of clinical course outcomes was assessed by using one-year post-device implantation complication rates such as stroke, gastrointestinal (GI) bleeds, DAT, and death.

To assess the difference in the rate of complications between the two groups (DAPT and OAC) and the association with gender, we conducted a two-sample Z-test of proportions using Prism statistical software. This test compares the proportion of patients who experienced complications in each group, and tests whether this proportion is influenced by gender. The test statistic is computed as follows: z=(p1−p2) ⁄√p∗(1−p)∗[ (1n⁄1)+(1n⁄2)]. 

P1 and p2 are the sample proportions of patients who experienced complications in the DAPT and OAC groups, respectively; n1 and n2 are the sample sizes of the two groups; and p is the pooled proportion of patients who experienced complications in both groups. The p-value is obtained by comparing the test statistic to the standard normal distribution. The P-value <0.05 indicates that the null hypothesis of no difference between the proportions can be rejected, meaning that there is a significant difference in the rate of complications between the two groups.

## Results

**Figure 1:**
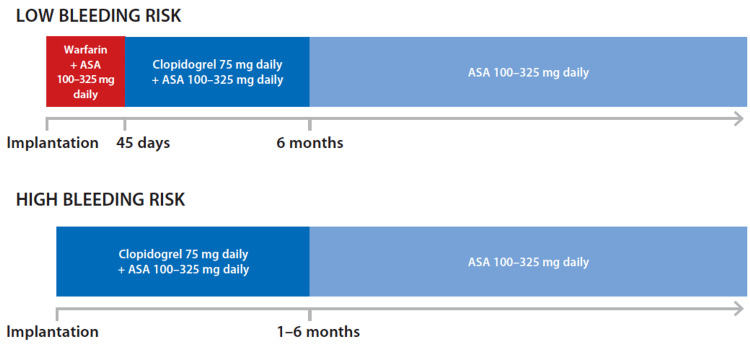
Timeline of antithrombotic treatment after left atrial appendage occlusion with the WATCHMAN™ device based on bleeding risk as recommended by the EHRA/EAPCI consensus statement EHRA, European Heart Rhythm Association; EAPCI, European Association of Percutaneous Coronary Interventions

Figure 1 depicts the timeline and composition of treatment after WATCHMAN™ implantation used in our study.

Our results from our 150-patient cohort revealed similarities and some differences between treatment regimens. As shown in Table 1, our patient makeup consisted of 101 (67.33%) males and 49 (32.60%) females. 74 (49.33%) patients were on OAC therapy compared to 54 (36.00%) on DAPT therapy. In the comparison of OAC therapy between genders, there was a greater proportion of males in this cohort, which was statistically significant (p=<0.01). No statistically significant difference was seen between genders on DAPT (p=0.22). There was also no significant difference in gender in the cohort that was not able to undergo implantation (p=0.08). Our results did reveal some concerns with WATCHMAN™ device implantation as 12 patients (8.00%) were not on any DAPT or OAC therapy during implant. Ten (6.66%) patients could not be implanted with the device, which is a higher-than-expected finding.

**Table 1:** Composition of participant gender and medication regimen OAC, oral anticoagulation; DAPT, dual antiplatelet therapy

Gender of patients	Frequency n (%)			
Males	101 (67.33)			
Females	49 (32.60)			
Drug regimen at implant		Male gender frequency (%)	Female gender frequency n (%)	P-value
OAC	74 (49.33)	54 (53.46)	20 (40.82)	<0.01
DAPT	54 (36.00)	31 (30.69)	23 (46.94)	0.22
None	12 (8.00)	9 (8.91)	3 (6.12)	0.08
Unsuccessful implant	10 (6.66)	7 (6.93)	3 (6.12)	0.19

In a comparison between complications, our results were encouraging for DAPT in place of OAC use. As shown in Table 2, there was no statistically significant difference in rates of ischemic stroke or DAT. Even though there was a higher number of GI bleeding in the DAPT cohort, this difference was not statistically significant (p=1.91). Overall, the rate of complications between the DAPT and OAC cohort were shown to be similar in our analysis. The 12 (8.00%) patients who were not placed on any medical therapy immediately post-device implantation did not suffer any ischemic cerebrovascular events in the 12-month period post-implant.

**Table 2:** Complications at 12 months post-WATCHMAN™ implantation OAC, oral anticoagulation; DAPT, dual antiplatelet therapy

Complications at 12 months	OAC cohort n (%)	DAPT cohort n (%)	P-value
Device-associated thrombus	2 (2.70)	0 (0)	0.22
Stroke	2 (2.70)	1 (1.85)	0.75
Gastrointestinal bleed	2 (2.70)	5 (9.26)	1.91
Complications	6 (8.11)	6 (11.11)	1.44

## Discussion

A key aspect of WATCHMAN™ device implantation is the choice of subsequent medical therapy consisting of either OAC or DAPT to reduce stroke risk and other complications [6]. Based on the 2019 American Heart Association/American College of Cardiology/Heart Rhythm Society (AHA/ACC/HRS) Focused Update of the 2014 AHA/ACC/HRS Guideline for the Management of Patients with Atrial Fibrillation, it is recommended to give aspirin (81-325 mg) with warfarin for 45 days post-WATCHMAN™ implantation. Warfarin is switched to clopidogrel (75 mg) after an absence of DAT and significant peri-device leak (jet width≤5 mm) on control TEE conducted around the 45th-day mark. Clopidogrel and aspirin are to be continued for up to six months post-procedure. Aspirin is continued indefinitely [7]. DAT is a key marker of any study regarding left atrial appendage closure with the WATCHMAN™ device. The exact mechanism of device-related thrombosis is unclear, but it likely involves incomplete endothelization of tissue surrounding the device, leading to thrombus formation. In a review of the PROTECT AF trial, transesophageal echocardiography revealed a DAT rate of 5.7% in patients who had undergone WATCHMAN™ implantation and were placed on OAC therapy for 45 days [8]. Compared to our study, this represents a higher rate of DAT in comparison to our OAC cohort (2.7%) and the DAPT cohort (0%). The PROTECT AF trial did have a significantly larger patient cohort of 707 patients and was conducted at 59 hospitals. Notably, it occurred from 2005 to 2008 and could be a consequence of the novelty of device implantation. A more recent review from 2008 to 2015 revealed a DAT rate of 3.9% in patients who had undergone WATCHMAN™ implantation and OAC medical therapy with 95% resolution of affected patients [9]. A meta-analysis of several large studies including the PROTECT AF trial examined the incidences of DAT between OAC and DAPT therapy, with results showing a higher incidence in DAPT therapy but treatment therapies revealed similar safety and efficacy endpoints [10]. DAT can increase the risk of neurological complications, but it is considered an unlikely complication of WATCHMAN™ implantation, which was seen in our study as well. Further research is needed to better understand the specific pathophysiology of DAT and identify any potential patient groups at increased risk of this complication [11].

An encouraging finding in our study was the limited incidence of stroke in both the OAC and DAPT patient cohorts. Given that the prevention of ischemic cerebral events is the treatment goal for WATCHMAN™ implantation, the success of this device in either treatment cohort in preventing these occurrences is paramount to the device’s continued use. The ASA Plavix feasibility study found comparable results to our DAPT cohort with all stroke episodes occurring in a small number of patients and made the case for DAPT being an effective alternative to OAC in the setting of WATCHMAN™ implantation [12]. Given that patients can have absolute contraindications to OAC use, it is critical to have well-studied alternatives to ensure adequate ischemic outcomes.

A concerning finding in our study was the rate of GI bleeding in the DAPT treatment group with five reported cases of GI bleeds even though this difference was not significant. Fortunately, in our cohort these five bleeding events were non-fatal. Given that a bleeding history could be the reason for many of the patients were placed on DAPT and not OAC, it is difficult to ascertain that DAPT was the cause for the increased bleeding risk. In the ASA Plavix feasibility study, the history of bleeding tendencies (93%) was the main contraindication for OAC use. The EWOLUTION study revealed the advanced age and comorbidities in the DAPT cohort showed a major bleeding rate of 2.1% excluding periprocedural events, which was significantly less than the expected Vitamin K antagonists HAS-BLED percentage of 5.1 [13]. Given the increased comorbidity and bleeding risk tendency of DAPT cohorts, DAPT appears to be a safe alternative in patients who cannot be placed on OAC therapy.

The strengths of our study included the 150 patient enrollment with a significant portion of DAPT patients. Given that all patients were followed at a single center and performed by the same team of two cardiac electrophysiologists, uniformity could be expected in the procedural aspects of WATCHMAN™ implantation as well as patient follow-up. A weakness in our study was the higher proportion of males included in the study, which was significant in the OAC cohort. Given that female gender is an independent risk factor for ischemic stroke in the setting of AF, our cohort results could have undervalued the potential risk of ischemic cerebral vascular events with OAC and DAPT treatments [14]. Since our study was only observational, our patients were not matched according to their CHA2DS2-VASc scores and HAS-BLED scores, two scoring systems used for stratifying clotting and bleeding risk, respectively. Patients with higher HAS-BLED scores carry higher bleeding risks, creating potential confounding variables as seen by other research in regard to DAPT use among higher-risk patients, especially in their GI bleeding propensity. Further research can also analyze the outcomes of NOAC use post-WATCHMAN™ implantation, given that these agents have decreased the risk of bleeding [15].

## Conclusions

Based on our single-center experience, our DAPT and OAC patient cohorts post-WATCHMAN™ device implantation fared similarly in a 12-month outcome analysis in regard to ischemic stroke and DAT. Although our DAPT cohort experienced a higher number of GI bleeding, it was not statistically significant in our analysis. GI bleeding is a common indication for patients not being placed on OAC and creates a possible confounding variable. Our work is in line with other larger studies that suggest DAPT is a safe and effective alternative to OAC therapy.
